# Unusual body weight loss due to primary hyperparathyroidism: A case study with literature review

**DOI:** 10.1016/j.heliyon.2024.e28333

**Published:** 2024-03-16

**Authors:** Yan-Yu Lin, Shuen-Fu Weng, Ting-Teng Yang, Yi-Wei Lee, Ju-Han Liu, Yu-Shan Hsieh

**Affiliations:** a Division of Endocrinology and Metabolism, Department of Internal Medicine, Taipei Medical University Hospital, Taipei City, 11031, Republic of China, Taiwan; b Division of Endocrinology and Metabolism, Department of Internal Medicine, School of Medicine, College of Medicine, Taipei Medical University, Taipei, Taiwan; c Department of Internal Medicine, Taipei Medical University Hospital, Taipei City, 11031, Republic of China, Taiwan; d School of Nursing, National Taipei University of Nursing and Health Sciences, Taipei City, 11230, Republic of China, Taiwan; e Department of Research, Taipei Medical University Hospital, Taipei City, 11031, Republic of China, Taiwan

**Keywords:** Hyperparathyroidism, Bubbly-like fracture, Brown tumor, Bone pain, Osteitis fibrosa cystica

## Abstract

Brown tumors (osteitis fibrosa cystica) are rare pathognomonic signs that occur in patients with primary hyperparathyroidism (PHPT). Brown tumors can exist in multiple bones and can easily be misdiagnosed as a metastatic tumor or multiple myeloma. It is also localized in the forearm, humerus, and leg. The symptoms of hypercalcemia, pathologic fracture, and bodyweight loss may increase the diagnostic difficulty of brown tumors because multiple myeloma and bone metastasis also show the same symptoms. We studied a 68-year-old woman who had experienced unusual bodyweight loss in the past 6 months (56kg–40kg) and bone pain. She went to the hospital after a fall with a complaint of bone pain. An X-ray revealed a left bubbly-like cystic change and multiple fractures at the left ulna midshaft. Upon investigation, the level of intact parathyroid hormone was ascertained to be 1800 (normal: 10–60) pg/ml. Microscopically, the tumor demonstrated a benign bone lesion and was compatible with osteitis fibrosa cystica due to PHPT. The parathyroid scan (Tc-99 m sestamibi) indicated right parathyroid hyperplasia, which was later confirmed by a parathyroidectomy. She was diagnosed with osteitis fibrosa cystica associated with PHPT due to a parathyroid adenoma. PHPT can be presented with multiple fractures, bone pain, and bodyweight loss. Therefore, if a patient presents these symptoms, PHPT should be considered.

## 1 Introduction

Primary hyperparathyroidism (PHPT) is a disorder in which excess parathyroid hormone (PTH) is secreted, and it is a symptomatic disorder commonly associated with hypercalcemia and bone disease. Incidences of PHPT are demonstrated in approximately 1 out of 2000 people every year. Among patients over 60 years old, it occurs twice as frequently in women [1].

Brown tumors (osteitis fibrosa cystica) are rare pathognomonic signs that occur in 1–4.5% of PHPT patients in clinical practice [2]. It is often localized and is a benign bony lesion caused by abnormal osteoclast activation of bone, resulting from direct effects of PHPT. The term “brown tumor” indicates hemosiderin accumulation then induces an abnormal bone lesion that has a macroscopically brown appearance [3]. There have been reports that most of them are benign tumors that usually affect the bones of the face [4]. In addition, brown tumors can exist in multiple bones and may easily be misdiagnosed as a metastatic tumor.

Most PHPT patients have been reported to have increased body weight because of greater insulin resistance and inhibited lipolysis [5]. Nevertheless, elevated intact parathyroid hormone (iPTH) levels could also trigger adipose tissue browning, which leads to increased energy expenditure and ultimately results in weight loss [6]. The symptoms of hypercalcemia, pathologic fracture, and bodyweight loss may increase the diagnostic difficulty of brown tumors because multiple myeloma and bone metastasis also show the same symptoms. In this case study, we present a 68-year-old woman that has an upper arm brown tumor and unusual bodyweight loss with PHPT due to parathyroid adenoma.

## 2 Case report

Our case study is on a 68-year-old woman who denied that she had a chronic disease. She had a history of left shoulder and right leg pain after falling 6 months prior and still felt pain and weakness. Moreover, unusual bodyweight loss in the past 6 months (56kg–40kg) with nausea and vomiting were noted.

**Fig. 1:**
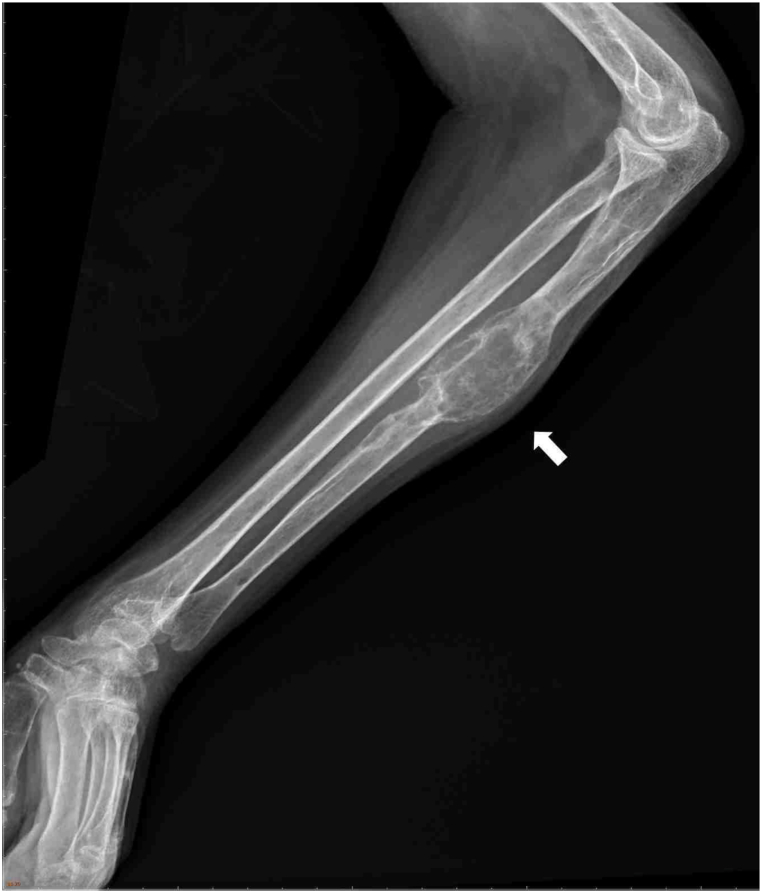
Bubbly-like cystic change and partial sclerotic margin at the left ulna midshaft metacarpal in X-ray.

Hence, she went to a hospital for emergency help. An X-ray examination revealed multifocal osteolytic lesions at the bilateral radius and the ulna appeared with larger bony expansile lesions with bubbly-like cystic change and partial sclerotic margin at the left ulna midshaft metacarpal (Fig. 1). The blood test results indicate an elevated white blood cell count (12.58 × 10^3/μl [4.00–11.00]). However, the ratios of neutrophils, lymphocytes, and monocytes remain within normal limits. The patient exhibits a slight reduction in hemoglobin levels (9.6 g/dl [[12], [13], [14], [15], [16]]) yet remains asymptomatic with no prior history of anemia. Notably, there's a minor elevation in the creatinine level (1.4 mg/dl [0.5–0.9]) and a small increase in the C-Reactive Protein level (0.57 mg/dl [<0.5]). Additionally, mild hypokalemia is observed (3.2 mEq/L [3.5–5.1]). Renal ultrasound findings reveal kidney stones measuring 1 cm in the right kidney and 0.9 cm in the left kidney, albeit without associated symptoms. Consequently, we have commenced symptomatic treatment to mitigate pain and gastrointestinal discomfort.

**Fig. 2:**
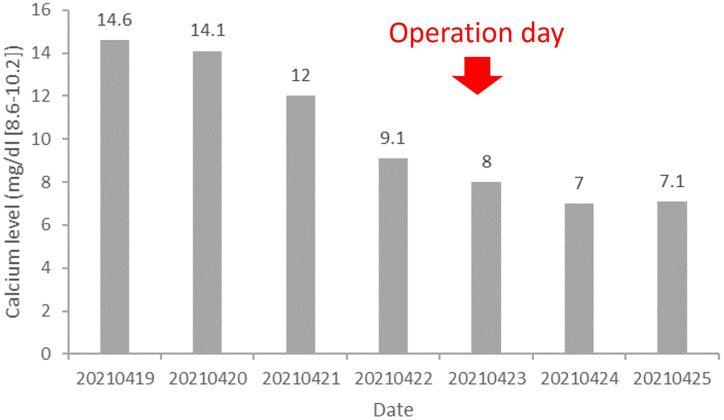
Changes in serum calcium levels during hospitalization.

**Fig. 3:**
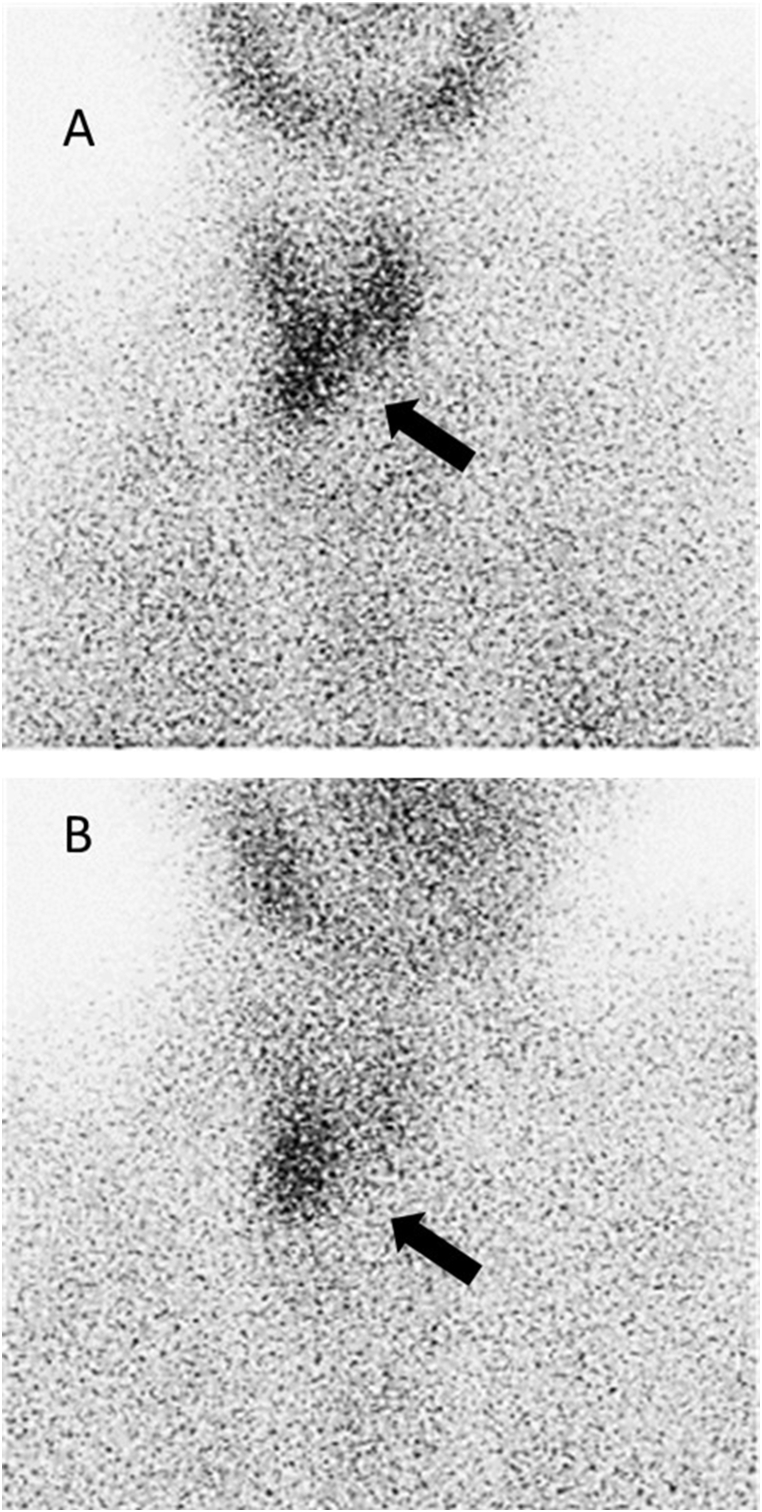
Right parathyroid hyperplasia (black arrow) was indicated by Tc-99 m sestamibi scan (A) 10min-early phase and (B) 3 hours-delayed phase.

In addition to evaluating the patient's primary concerns of unexplained weight loss, bone pain, and weakness, we also assessed tumor markers for multiple myeloma and other malignant neoplasms. Our investigation revealed no abnormalities in tumor marker levels, including Carcinoembryonic Antigen (CEA), Cancer Antigen 199 (CA199), and the Immunoglobulin G (IgG) kappa/lambda ratio. However, the intact parathyroid hormone (iPTH) level was significantly elevated at 1800 pg/ml (normal range: 10–60 pg/ml). The serum calcium level was also notably high at 14.6 mg/dl (normal range: 8.6–10.2 mg/dl), and the phosphate level was slightly below the normal range at 2.2 mg/dl (normal range: 2.7–4.5 mg/dl) (see Fig. 2). Results from the dual-energy X-ray absorptiometry (DXA) scan indicated T-scores of −4.6 and −4.9 at the left hip and lumbar spine, respectively. Echography findings identified two extrathyroidal lesions behind the right thyroid lobe, suggestive of enlarged parathyroid glands. These results indicated that the hypercalcemia was associated with hyperparathyroidism. Microscopically, the bubbly-like cystic bony lesions showed a benign bone lesion with blood-filled cystic spaces separated by fibrous septa containing multinucleated osteoclast-type giant cells and bland spindle cells, which is compatible with osteitis fibrosa cystica. Because two enlarged parathyroid glands behind the right thyroid bed were found by sonography, we arranged a parathyroid scan (Tc-99 m sestamibi; MIBI), which indicated right parathyroid hyperplasia (Fig. 3) due to PHPT.

After a discussion with the patient and the family, parathyroidectomy surgery was performed. A 2.8 × 2.5 × 1.8 cm (weight:5.41g) parathyroid gland was found at the lower right neck and was suspected to be adenoma; other parathyroid glands were a normal size. The day after surgery, the symptoms of nausea and vomiting resolved dramatically. The level of iPTH was reduced to 127 pg/ml and the serum calcium level was 7 mg/dl, alkaline phosphatase level was high (382 U/L [35−104]). Histologically, the lesion was composed of non-neoplastic parathyroid adenoma (pathologic diagnosis: Presence of non-neoplastic parathyroid tissue and adenoma). Considering the likelihood of persistent hungry bone syndrome for several days following surgery, we prescribed the patient oral calcium carbonate at a dose of 3 mg/day. This was accompanied by ongoing monitoring of serum calcium levels for the first 7 days, after which we switched to oral calcitriol at 0.5 μg/day. At the outpatient follow-up, conducted 14 days post-procedure, the patient's serum calcium levels had shown gradual improvement, reaching 7.3 mg/dl [normal range: 8.6–10.2 mg/dl].

## 3 Discussion

PHPT is chiefly caused by adenoma or chief cell hyperplasia of the parathyroid gland. Clinical manifestations of parathyroid adenoma include hypercalcemia and bone deformities with fractures, which might be the initial presentation of primary hyperparathyroidism (parathyroid adenoma). Brown tumors are rare pathognomonic signs of hyperparathyroidism localized in the forearm, humerus, and leg. Its name comes from its characteristic color resulting from its hemosiderin deposition. A brown tumor is a characteristic focal osteolytic lesion that is seen in PHPT, and it is filled with many osteoclasts, macrophages, and fibroblasts. The brown appearance is due to its high vascularity with its concomitant hemorrhage and hemosiderin deposition. Patients may present symptoms such as bone pain, fractures, bodyweight loss, and weakness, which might lead to a misdiagnosis of a malignant tumor or multiple myeloma.

**Fig. 4:**
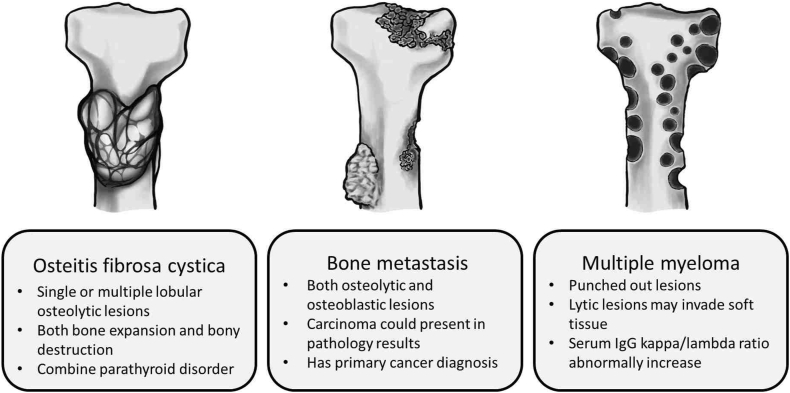
The lesion was composed of non-neoplastic parathyroid adenoma in histological result.

In addition, Brown tumors can exist in multiple bones and may easily be misdiagnosed as metastatic tumors because of multiple fractures [7]. In fact, brown tumors, multiple myeloma, and bone metastasis all have different characteristics. Brown tumors mainly consist of single or multiple lobular osteolytic lesions, usually with bone expansion and bony destruction [8]. Lytic lesions are the main finding of multiple myeloma, and are described as “punched out lesions”; results in previous studies indicate that bone lytic lesions may invade soft tissue [9]. Bone metastasis might have both osteolytic and osteoblastic lesions present in breast, gastrointestinal, or other squamous cancers [10]. The characteristics are presented in Fig. 4.

PHPT has been reported to be associated with increased incidences of dyslipidemia, hypertension, weight gain, impaired glucose tolerance, diabetes mellitus, and mortality [ [11,12]]. Most PHPT patients have been reported to have increased body weight because of greater insulin resistance and inhibited lipolysis [5]. However, secondary hyperparathyroidism seems to demonstrate another mechanism of wasting, especially among chronic dialysis patients; bodyweight loss might be a mediator between elevated PTH levels and mortality [13]. In this study, a patient was diagnosed with PHPT with bodyweight loss.

Elevated PTH levels have been known to induce adipose tissue browning, which increases energy expenditure and can consequently lead to weight loss, as observed in our patient [6]. Moreover, we noted that hyperglycemia and weakness induced by PHPT contributed to a decreased appetite, further exacerbating the weight loss. Previous studies have indicated that gastrointestinal symptoms associated with hypercalcemia, primarily presenting as anorexia, nausea, and vomiting, often result in reduced food intake and subsequent weight loss. It has been documented that in patients with primary hyperparathyroidism (PHPT), normalizing calcium ion levels typically leads to an improvement in appetite, increased food intake, and eventual weight gain [14]. However, in this case study, despite an improvement in gastrointestinal symptoms following the 14-day postoperative period, there was no significant increase in the patient's weight (from 40kg to 41kg). This suggests that further monitoring may be necessary to fully understand the patient's weight dynamics post-treatment.

Previous research has highlighted that primary hyperparathyroidism (PHPT) and weight loss are directly influenced by the action of a parathyroid hormone-related protein (PTHrP), which stimulates the browning of white adipose tissue (WAT) [15]. Additionally, studies have shown that elevated parathyroid hormone (PTH) levels, leading to weight loss, may influence mortality rates in patients with secondary hyperparathyroidism [16] or in those undergoing long-term dialysis in the absence of parathyroid diseases [17]. However, there is limited research exploring the relationship between PTH levels, weight, and mortality rates specifically in patients with primary hyperparathyroidism (PHPT). Future studies should aim to elucidate how PTH levels impact long-term outcomes in these patients.

Our study is subject to certain limitations. Firstly, detailed reports on the patient's food and drink intake were not available, which leaves open the possibility of reduced food consumption contributing to the weight loss. Secondly, although we investigated to rule out secondary hyperparathyroidism, weight loss can also be a symptom of other medical conditions. Thirdly, our study lacks a comprehensive follow-up on the patient's body weight variations post-surgery. If the weight loss was indeed a result of primary hyperparathyroidism (PHPT), an improvement in body weight should have been evident post-treatment. Finally, this case presented with 'hungry bone syndrome,' a condition marked by sustained and severe hypocalcemia typically seen after parathyroidectomy and thyroidectomy. Managing this hypocalcemic state is challenging and often requires varying doses of supplements to mitigate patient morbidity. This syndrome is due to the sudden drop in parathyroid hormone (PTH) levels and its effects on osteoclastic resorption, with reduced PTH leading to unopposed osteoblast activation and a surge in calcium influx into the 'calcium-starved' bones. The prolonged preoperative elevation of PTH levels is directly correlated with the severity of post-parathyroidectomy hypocalcemia [18]. Prior research suggests treating hungry bone syndrome with 6–12 g/day of elemental calcium. Moreover, administering 2–4 mg/day of calcitriol both preoperatively and postoperatively is highly recommended, as it has been effective in maintaining serum calcium levels following surgery [19]. The need for calcium supplementation post-surgery to prevent complications is clear. Further research could explore the impact of hungry bone syndrome incidence on long-term body weight outcomes.

In conclusion, this report presents a rare case of a patient diagnosed with PHPT accompanied by a brown tumor, multiple fractures, and significant weight loss. Brown tumors, which can occur in various bones, are often easily misinterpreted as metastatic tumors due to the presence of multiple fractures. Additionally, the weight loss observed in this patient could have led to a misdiagnosis of other malignant conditions. This case underlines the possibility that weight loss can be a presenting symptom of PHPT, contributing to the understanding of its clinical manifestations.

## Data availability statement

The data associated with the study has not been deposited into any publicly available repository. Data will be made available on request.

## Ethical approval

Research Ethics Committee of Institutional Review Board of Taipei Medical University (Approval No: N202206038). Clinical participant provided informed consent.

## Funding

None.

## CRediT authorship contribution statement

**Yan-Yu Lin:** Project administration, Methodology, Investigation, Formal analysis, Data curation. **Shuen-Fu Weng:** Writing – review & editing, Supervision, Software, Methodology. **Ting-Teng Yang:** Writing – review & editing. **Yi-Wei Lee:** Writing – review & editing. **Ju-Han Liu:** Writing – review & editing, Conceptualization. **Yu-Shan Hsieh:** Writing – review & editing, Writing – original draft, Visualization, Validation, Conceptualization.

## Declaration of competing interest

The authors declare that they have no known competing financial interests or personal relationships that could have appeared to influence the work reported in this paper.
